# Laboratory, Clinical, and Survival Outcomes Associated With Peptide Receptor Radionuclide Therapy in Patients With Gastroenteropancreatic Neuroendocrine Tumors

**DOI:** 10.1001/jamanetworkopen.2021.2274

**Published:** 2021-03-23

**Authors:** Sarit T. Kipnis, Matthew Hung, Shria Kumar, Jason M. Heckert, Hwan Lee, Bonita Bennett, Michael C. Soulen, Daniel A. Pryma, David A. Mankoff, David C. Metz, Jennifer R. Eads, Bryson W. Katona

**Affiliations:** 1 Department of Medicine, Hospital of the University of Pennsylvania, Philadelphia; 2 Department of Radiology, Perelman School of Medicine, University of Pennsylvania, Philadelphia; 3 Division of Gastroenterology, Perelman School of Medicine, University of Pennsylvania, Philadelphia; 4 Division of Hematology and Oncology, Perelman School of Medicine, University of Pennsylvania, Philadelphia

## Abstract

**Importance:**

Peptide receptor radionuclide therapy (PRRT) is approved in the US for treatment of gastroenteropancreatic neuroendocrine tumors (NETs), but data on PRRT outcomes within US populations remain scarce.

**Objective:**

To analyze the first 2 years of PRRT implementation at a US-based NET referral center.

**Design, Setting, and Participants:**

This cohort study was conducted using medical records of patients with metastatic NET receiving PRRT from 2018 through 2020 in a NET program at a tertiary referral center. Included patients were those at the center with metastatic NETs who received at least 1 dose of PRRT over the study period. Laboratory toxic effects were assessed using Common Terminology Criteria for Adverse Events version 5.0. Tumor response was determined using Response Evaluation Criteria in Solid Tumors 1.1. Survival analysis was conducted to identify factors associated with progression-free survival (PFS) and overall survival. Data were analyzed from August 2018 through August 2020.

**Exposures:**

Receiving 4 cycles of lutetium-177-dotatate infusion, separated by 8-week intervals targeted to 7.4 GBq (200 mCi) per dose.

**Main Outcomes and Measures:**

Data were compared from before and after PRRT to determine hematologic, liver, and kidney toxic effects and to assess tumor progression and patient survival.

**Results:**

Among 78 patients receiving at least 1 dose of PRRT, median (interquartile range) age at PRRT initiation was 59.8 (53.5-69.2) years and 39 (50.0%) were men. The most common primary NET sites included small bowel, occurring in 34 patients (43.6%), and pancreas, occurring in 22 patients (28.2%). World Health Organization grade 1 or 2 tumors occurred in 62 patients (79.5%). Among all patients, 56 patients underwent pretreatment with tumor resection (71.8%), 49 patients received nonsomatostatin analogue systemic therapy (62.8%), and 49 patients received liver-directed therapy (62.8%). At least 1 grade 2 or greater toxic effect was found in 47 patients (60.3%). Median PFS was 21.6 months for the study group, was not reached by 22 months for patients with small bowel primary tumors, and was 13.3 months for patients with pancreatic primary tumors. Having a small bowel primary tumor was associated with a lower rate of progression compared with having a pancreatic primary tumor (hazard ratio, 0.19; 95% CI, 0.07-0.55; P = .01). Median overall survival was not reached.

**Conclusions and Relevance:**

This cohort study of patients with metastatic NETs found that PRRT was associated with laboratory-measured toxic effects during treatment for most patients and an overall median PFS of 21.6 months. Patients with small bowel NETs had longer PFS after PRRT compared with patients with pancreatic NETs.

## Key Points

**Question:**

What are the clinical outcomes for patients who receive peptide receptor radionuclide therapy (PRRT) for neuroendocrine tumors (NETs) in US-based populations?

**Findings:**

In this cohort study of 78 patients who underwent PRRT, the treatment was associated with transient laboratory-measured toxic effects in most patients. Median progression-free survival was significantly increased for patients with small bowel primary tumors compared with patients with pancreatic primary tumors.

**Meaning:**

These findings suggest that PRRT may be a useful treatment for NETs in US-based populations. The study found that patients with small bowel NETs have significantly longer progression-free survival compared with patients with pancreatic NETs.

## Teaser

This cohort study examines laboratory, clinical, and survival outcomes among patients with gastroenteropancreatic neuroendocrine tumors (NETs) treated with peptide receptor radionuclide therapy.

## Introduction

Neuroendocrine tumors (NETs) are a heterogeneous group of neoplasms, with primary tumors commonly originating in the pancreas, intestines, and lungs.^1,2,3^ These tumors often present with distant metastatic disease, and given the multitude of treatment options available, treatment of metastatic disease is growing increasingly complex.^4^ Somatostatin analogues are widely used to treat metastatic NETs and have been shown to slow progression and improve disease-related symptoms.^5,6^ Other potential therapies for metastatic NETs include systemic chemotherapy, liver-directed therapy, surgical treatment, and peptide receptor radionuclide therapy (PRRT).^4,7,8,9^

In PRRT, a radionuclide linked to a somatostatin analogue is used, allowing for targeted delivery of radiotherapy to somatostatin receptor–expressing NETs, which represent most NETs.^10,11^ The first randomized clinical trial assessing the efficacy of PRRT for treatment of NETs was the Neuroendocrine Tumors Therapy (NETTER-1) trial, which included patients with metastatic, well-differentiated (ie, World Health Organization [WHO] grade 1 or 2) midgut NETs who had progressed on somatostatin analogue therapy.^12^ In this landmark phase 3 multicenter trial, patients were randomized to receive lutetium-177-dotatate plus octreotide long-acting repeatable or high-dose octreotide long-acting repeatable alone. Progression-free survival (PFS) was significantly higher in the lutetium-177-dotatate group than in the control group. After results of NETTER-1 supported the effectiveness of PRRT in midgut NETs, the Food and Drug Administration (FDA) approved lutetium-177-dotatate for treatment of somatostatin receptor–positive gastroenteropancreatic NETs in 2018. Since FDA approval of PRRT, efforts have been targeted at implementation of PRRT within NET clinics across the United States.^13,14^ Results from a 2020 study^15^ by our group suggested that PRRT could be successfully administered among a diverse US-based group of patients with NETs, and we found that patients with abnormal liver function tests before PRRT had an increased likelihood of discontinuing PRRT prior to completion of the PRRT treatment course.

Prior to FDA approval of PRRT, and apart from NETTER-1, multiple studies^16,17,18,19^ reported outcomes of PRRT in patients with NETs. However, to our knowledge, there have been no studies after PRRT was approved describing PRRT outcomes for patients with NETs who underwent treatment in the United States. This unique population of patients received a treatment course that may have differed from that in other parts of the world owing to the delayed PRRT approval in the US and represent a more heterogeneous population than patients enrolled in NETTER-1. Given the increased accessibility to PRRT and increased use of the treatment within the US, it is critical to understand clinical outcomes to establish expectations for patients and clinicians. In this study, we examined the first 2 years of PRRT implementation within a tertiary US NET referral center; we describe the patient group selected for PRRT and examine their laboratory-measured toxic effects and therapeutic response.

## Methods

This cohort study was approved through the University of Pennsylvania Institutional Review Board, which granted a waiver of informed consent for participation because this study was deemed to be of minimal risk. This study is reported following the Strengthening the Reporting of Observational Studies in Epidemiology (STROBE) reporting guideline.

We collected clinical data from patients with metastatic NETs who underwent PRRT through the University of Pennsylvania NET Program during the first 2 years of PRRT implementation (ie, August 2018 through August 2020). All patients who had received at least 1 dose of PRRT at our institution were included in the study, including 4 patients who received a portion of PRRT at a different institution. Our institution follows current guidelines in which a standard course of PRRT includes 4 doses of lutetium-177-dotatate, separated by 8-week intervals and targeted to 7.4 GBq (200 mCi) per dose.

Electronic medical records of all patients included in the study were reviewed. Study-related data included sex, date of birth, date of death (if applicable), date of NET diagnosis, primary tumor location, primary tumor grade, and presence of liver metastases. Prior treatment data was collected, including use of somatostatin analogue, use of systemic chemotherapy, resection of primary tumor, and use of liver-directed therapy, including transarterial chemoembolization (TACE), transarterial radioembolization (TARE), bland embolization, radiofrequency ablation (RFA), and hepatic resection. Baseline laboratory data was collected, including hemoglobin level, white blood cell count, platelet count, creatinine level, estimated glomerular filtration rate, total bilirubin level, aspartate aminotransferase level, and alanine aminotransferase level. Subsequent laboratory values obtained between doses of PRRT were also recorded.

Laboratory measures of toxic effects were determined using Common Terminology Criteria for Adverse Events version 5.0 from the National Institutes of Health National Cancer Institute^20^ and were defined as the development of new grade 2 or higher laboratory-measured toxic effect during or after PRRT within the study period (eAppendix in the Supplement). Laboratory values were recorded after each PRRT dose and compared with pre-PRRT laboratory measures. Toxic effect measures were counted if they were new compared with pre-PRRT measures to account for baseline laboratory abnormalities. Patients with ongoing or worsening laboratory abnormalities were counted in subsequent cycles if they continued to receive PRRT. Grade 3 or 4 toxic effects (eAppendix in the Supplement) were assessed and were counted regardless of laboratory abnormalities before PRRT initiation to capture patients who developed worsening laboratory abnormalities.

Response Evaluation Criteria in Solid Tumors (RECIST) 1.1^21^ was used to determine progression between pre-PRRT and post-PRRT imaging studies, which included contrast-enhanced computed tomography and contrast-enhanced magnetic resonance imaging. The most recent cross-sectional imaging prior to the first dose of PRRT served as the baseline for the patient's radiologic burden of disease. All images obtained after the patient had received at least 1 PRRT dose were reviewed and evaluated for progression. Patients who had not completed 4 doses of PRRT were also included in the analysis in addition to patients who had completed 4 doses; this included patients who delayed or discontinued therapy. Data were analyzed by comparing individuals who progressed with those in the remaining study group. Given that some individuals did not yet have post-PRRT imaging, a subanalysis was performed to compare individuals who progressed with those with post-PRRT imaging who did not progress.

### Statistical Analysis

Statistical analysis was performed using Stata/IC statistical software version 15.1 (StataCorp). Comparative statistical analyses were performed, and PFS and overall survival were analyzed using Cox proportional hazards models. Kaplan-Meier plots were produced. *P* values were calculated using log-rank test for survival curves. For PFS, events were defined by progression or death. Patients were censored at end of follow-up. Overall survival included death by any cause. Univariable analysis was performed to identify factors associated with survival. All statistical tests were 2-tailed, and significance was defined as *P* < .05.

## Results

### Cohort Characteristics

Among 78 patients who received at least 1 dose of PRRT during the study period, the median (interquartile rage [IQR]) age was 53.7 (48.0-62.4) years at NET diagnosis and 59.8 (53.5-69.2) years at first PRRT; 39 (50.0%) patients were men (Table 1). There was a diversity in primary NET location, with 34 small bowel tumors (43.6%), 22 pancreas tumors (28.2%), and 22 primary tumors in other locations, including colon, stomach, lung, and ovary. The World Health Organization tumor grade of the NET was 1 in 27 patients (34.6%), 2 in 35 patients (44.9%), 3 in 8 patients (10.3%), and unknown in 8 patients (10.3%). Somatostatin analogue prior to PRRT was used in all but 1 patient (98.7%). In the study group, 49 patients (62.8%) underwent nonsomatostatin analogue systemic chemotherapy prior to PRRT and 49 patients underwent liver-directed therapy prior to PRRT, including 21 patients with hepatic resections (26.9%), 7 patients with radiofrequency ablation (9.0%), 14 patients with bland embolization (17.9%), 16 patients with transarterial chemoembolization (20.5%), and 14 patients with transarterial radioembolization; 56 patients (71.8%) had the primary tumor resected.

**Table 1.** Patient Characteristics

Characteristic	No. (%)			*P* value^a^
	Total (N = 78)	With no progression (n = 53)	With progression (n = 25)	
Age at diagnosis, median (IQR), y	53.7 (48.0-62.4)	53.8 (48.0-65.3)	52.6 (49.0-58.8)	.57
Age at first PRRT, median (IQR), y	59.8 (53.5-69.2)	59.9 (55.2-70.0)	58.2 (53.4-66.6)	.19
Sex				
Men	39 (50.0)	28 (52.8)	11 (44.0)	.47
Women	39 (50.0)	25 (47.2)	14 (56.0)	
NET primary location				
Pancreas	22 (28.2)	10 (18.9)	12 (48.0)	.007
Small bowel	34 (43.6)	29 (54.7)	5 (20.0)	
Other or unknown	22 (28.2)	14 (26.4)	8 (32.0)	
Grade of NET				
1	27 (34.6)	21 (39.6)	6 (24.0)	.11
2	35 (44.9)	25 (47.2)	10 (40.0)	
3	8 (10.3)	4 (7.5)	4 (16.0)	
Unknown	8 (10.3)	3 (5.7)	5 (20.0)	
Systemic therapy prior to PRRT	49 (62.8)	33 (62.3)	15 (60.0)	.85
Liver-directed therapy prior to PRRT	49 (62.8)	35 (66.0)	14 (56.0)	.39
Hepatic resection	21 (26.9)	14 (26.4)	7 (28.0)	.88
Radiofrequency ablation	7 (9.0)	6 (11.3)	1 (4.0)	.29
Bland embolization	14 (17.9)	11 (20.8)	3 (12.0)	.35
Transarterial chemoembolization	16 (20.5)	12 (22.6)	4 (16.0)	.50
Transarterial radioembolization	14 (17.9)	10 (18.9)	4 (16.0)	.76
Prior resection of primary tumor	56 (71.8)	39 (73.6)	17 (68.0)	.61

Abbreviations: IQR, interquartile range; NET, neuroendocrine tumor; PRRT, peptide receptor radionuclide therapy.

^a^ *P* value compares no progression vs progression groups.

**Figure 1.**
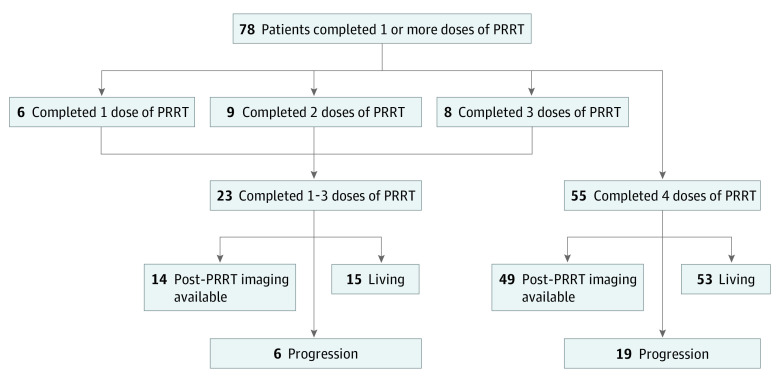
Peptide Receptor Radionuclide Therapy (PRRT) Study Group

Median (IQR) duration of follow-up was 15.5 (8.7-19.8) months. Of 78 patients who received at least 1 dose of PRRT, 55 patients (70.5%) had completed all 4 doses at the time of the analysis (Figure 1). Of 23 patients (29.5%) who had not completed 4 doses at the time of analysis, 6 patients had completed 1 dose of PRRT, 9 patients had completed 2 doses, and 8 patients had completed 3 doses. Reasons for not completing therapy included death among 8 patients, as well as progression, toxic effects, and other events among 7 patients. The remaining 8 patients intended to receive 4 doses at the time of the analysis.

### Laboratory-Measured Toxic Effects

At least 1 new grade 2 or greater laboratory-measured toxic effect was found in 47 patients (60.3%) during their treatment course (Table 2). The most common new grade 2 or greater laboratory-measured toxic effect was leukopenia, found in 26 patients (33.3%), followed by anemia, found in 16 patients (20.5%); acute kidney injury (AKI), found in 12 patients (15.4%); liver injury, found in 12 patients; and thrombocytopenia, found in 9 patients (11.5%). Of 12 patients with AKI, 6 patients had non-PRRT–related causes of kidney injury documented in the medical record. Among 55 patients who completed 4 doses of PRRT, the most common toxic effect after the final dose was leukopenia, found in 13 patients (23.6%), followed by thrombocytopenia, found in 7 patients (12.7%); anemia, found in 6 patients (10.9%); AKI, found in 3 patients (5.5%); and liver injury, found in 3 patients. Grade 3 or 4 laboratory-measured toxic effects were observed in 25 patients (32.1%) throughout treatment. Among all patients in the study, the most common grade 3 or 4 laboratory-measured toxic effects were anemia, found in 10 patients (12.8%), and leukopenia, found in 10 patients. Grade 3 or 4 thrombocytopenia was found in 7 patients (9.0%). While 1 patient developed transfusion-dependent anemia, this patient had baseline anemia associated with prior treatment with everolimus. No one in the study group developed myelodysplastic syndrome, dialysis-dependent kidney injury, or liver failure. Dosage was reduced for 2 patients owing to laboratory-measured toxic effects.

**Table 2.** Laboratory-Measured Toxic Effects During and After Treatment^a^

	No. (%)				
	Dose 1 (n = 78)	Dose 2 (n = 72)	Dose 3 (n = 63)	Dose 4 (n = 55)	Total (n = 78)
Any grade 2-4 toxic effect					
Any toxic effect	14 (18.0)	29 (40.3)	21 (33.3)	21 (38.2)	47 (60.3)
Anemia	1 (1.3)	11 (15.3)	4 (6.4)	6 (10.9)	16 (20.5)
Leukopenia	6 (7.7)	17 (23.6)	15 (23.8)	13 (23.6)	26 (33.3)
Thrombocytopenia	2 (2.6)	4 (5.6)	4 (6.4)	7 (12.7)	9 (11.5)
Acute kidney injury	5 (6.4)	5 (6.9)	1 (1.6)	3 (5.5)	12 (15.4)
Liver injury	3 (3.8)	6 (8.3)	2 (3.2)	3 (5.5)	12 (15.4)
Grade 3 or 4 toxic effect^b^					
Any toxic effect	6 (7.7)	8 (11.1)	7 (11.1)	12 (21.8)	25 (32.1)
Anemia	0 (0.0)	6 (8.3)	1 (1.6)	5 (9.1)	10 (12.8)
Leukopenia	1 (1.3)	3 (4.2)	5 (7.9)	5 (9.1)	10 (12.8)
Thrombocytopenia	2 (2.6)	3 (4.2)	0 (0.0)	4 (7.3)	7 (9.0)
Acute kidney injury	1 (1.3)	2 (2.8)	2 (3.2)	0 (0.0)	5 (6.4)
Liver injury	3 (3.8)	1 (1.4)	0 (0.0)	1 (1.8)	6 (7.7)

^a^ Patients with ongoing or worsening laboratory-measured toxic effects were counted in future doses if they continued to receive peptide receptor radionuclide therapy.

^b^ Patients with grade 3 to 4 toxic effects were counted regardless of laboratory abnormalities before peptide receptor radionuclide therapy.

### PFS

**Figure 2.**
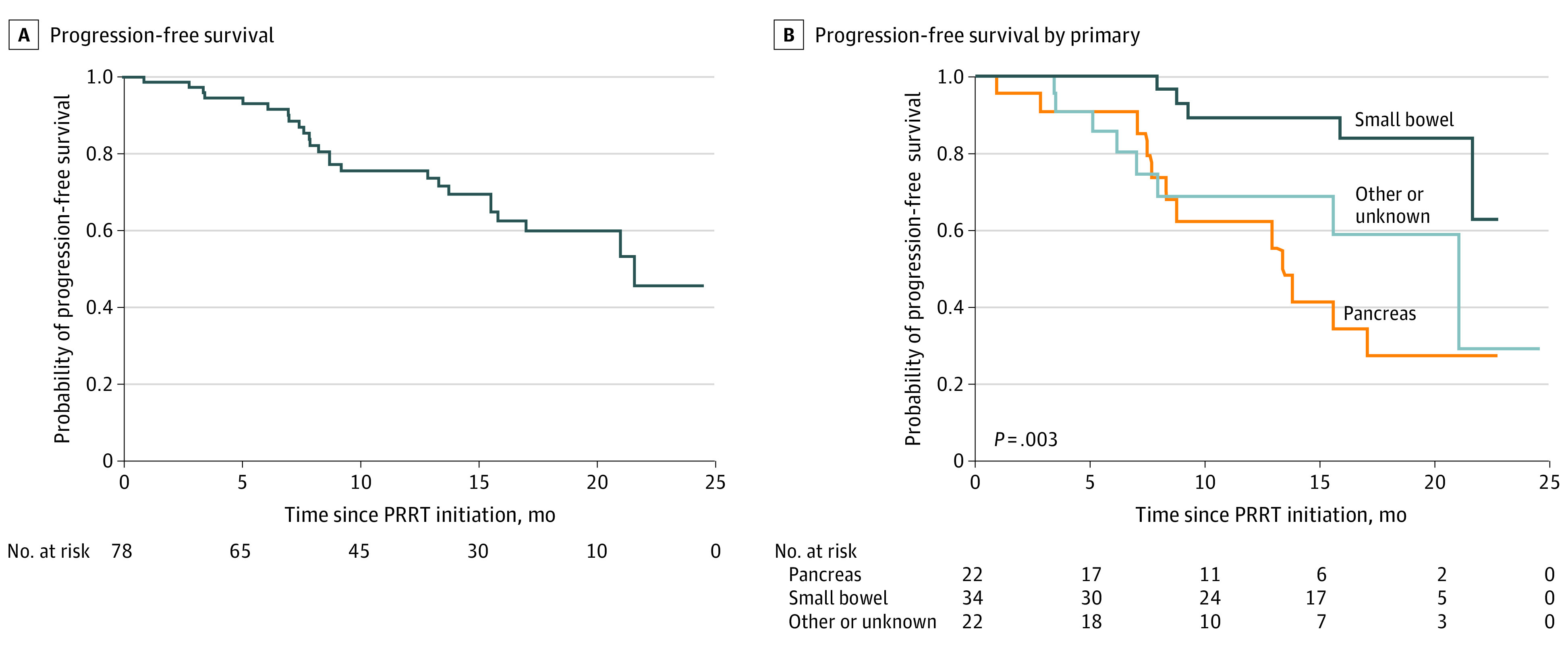
Progression-Free Survival After Peptide Receptor Radionuclide Therapy (PRRT)

During the study period, 30 patients (38.5%) had progression or died, 25 patients (32.1%) had evidence of progression on imaging, and 5 patients (6%) who did not have progression on imaging died during the treatment course. The only significantly different characteristic between patients who progressed and those who did not was NET primary location (Table 1). Median PFS for the entire study group was 21.6 months (Figure 2, part A). Univariable analysis of factors associated with progression is shown in Table 3. Having a small bowel NET was associated with a lower rate of progression compared with having a pancreatic NET (hazard ratio [HR], 0.19; 95% CI, 0.07-0.55; P = .01) (Table 3). Similar results were obtained when restricting the analysis to patients who had analyzable imaging after PRRT (eTable 1 in the Supplement). By 22 months, median PFS was not reached for patients with small bowel NETs, whereas PFS was 13.3 months for patients with pancreatic NETs and 21.0 months for patients with other or unknown primary NETs (*P* = .003) (Figure 2, part B). Age, sex, tumor grade, prior systemic therapy, prior liver-directed therapy, and prior resection of primary tumor were not associated with statistically significant differences in progression.

**Table 3.** Univariable Analysis of Factors Associated With Progression After PRRT

	HR (95% CI)	*P* value
Age at diagnosis, median (IQR), y	1.00 (0.86-1.04)	.92
Age at first PRRT, median (IQR), y	0.99 (0.95-1.03)	.49
Men	0.76 (0.34-1.69)	.50
NET primary location		
Pancreas	1 [Reference]	NA
Small bowel	0.19 (0.07-0.55)	.01
Other or unknown	0.68 (0.28-1.66)	.39
Grade of NET		
1	1 [Reference]	NA
2	1.13 (0.41-3.12)	.81
3	2.57 (0.72-9.17)	.15
Systemic therapy prior to PRRT	1.13 (0.51-2.52)	.77
Liver-directed therapy prior to PRRT	0.71 (0.32-1.57)	.40
Hepatic resection	0.90 (0.38-2.16)	.81
Radiofrequency ablation	0.53 (0.07-3.92)	.53
Bland embolization	0.88 (0.26-2.99)	.84
Transarterial chemoembolization	0.69 (0.24-2.02)	.50
Transarterial radioembolization	0.90 (0.31-2.63)	.85
Prior resection of primary tumor	0.76 (0.33-1.78)	.53

Abbreviations: HR, hazard ratio; IQR, interquartile range; NA, not applicable; NET, neuroendocrine tumor; PRRT, peptide receptor radionuclide therapy.

### Overall Survival

In this study group, 10 patients (12.8%) died after completion of at least 1 dose of PRRT; however, given the short follow-up period, median overall survival was not reached (eFigure in the Supplement). Additionally, 2 patients (2.6%) died after completion of 1 cycle of treatment, 4 patients (5.1%) died after completion of 2 cycles, 2 patients died after completion of three cycles; and 2 patients died after completion of 4 cycles. Univariable analysis of factors associated with survival was significant for age at NET diagnosis (HR, 1.08; 95% CI, 1.01-1.16; *P* = .02) (eTable 2 in the Supplement).

## Discussion

This cohort study of outcomes associated with PRRT in a non-clinical trial–based US NET group after FDA approval of PRRT found that patients with small bowel primary tumors had significantly lower rates of progression compared with patients with pancreatic primary tumors.

One strength of our study is that our NET group was diverse and heterogeneous, which may provide a realistic representation of clinical NET practices. Our study group was heavily pretreated prior to PRRT initiation, unlike the NETTER-1 population. While pretreatment was not found to be associated with progression after PRRT, studies with larger study groups or longer follow-up periods may find pretreatment associations with treatment outcomes. A 2017 study^16^ by our group found that prior systemic chemotherapy was a predictor associated with decreased PFS, although this study had a longer follow-up period after PRRT. Ultimately, given the number of treatment options for NETs, more studies focused on the sequence of therapies may help elucidate where best to incorporate PRRT into the NET treatment algorithm.

Our study also differed from NETTER-1 in primary tumor location and grade. Whereas NETTER-1 was restricted to patients with midgut NETs,^12^ our patient population had NETs arising from locations outside of the small bowel, including the pancreas, colon, stomach, and lung, which allowed us to compare PFS among the primary tumor sites. Additionally, NETTER-1 included only patients with grade 1 or 2 NETs, while more than 10% of patients in our group had grade 3 NETs. The NETTER-2 trial will provide critical information that may address efficacy of PRRT in non-small bowel and advanced-grade NETs^22^; however, results from that study will not be available for several years.

While PRRT is generally accepted as a well-tolerated treatment for NETs, 47 out of 78 patients in our study (60.3%) experienced a laboratory-measured toxic effect at some point during their treatment course. Laboratory-measured toxic effects within our study group were similar to those reported in 3 studies from 2019,^13,23,24^ and included transient leukopenia, anemia, thrombocytopenia, AKI, and liver injury. Compared with the NETTER-1 population, a greater proportion of our patients experienced anemia (16 patients [20.5%] in our study group vs 16 patients [14.4%] in NETTER-1) and leukopenia (26 patients [33.3%] in our study group vs 11 patients [9.9%] in NETTER-1), but a smaller proportion of our patients experienced thrombocytopenia (9 patients [11.5%] in our study group vs 28 patients [25.2] in NETTER-1).^12^ While 15% of our study group experienced AKI, half of these patients had a non-PRRT–related cause of AKI documented, suggesting that the rate of AKI associated with PRRT is likely lower than reported in this study. Additionally, a greater proportion of patients in our study group experienced grade 3 or 4 toxic effects compared with patients in NETTER-1: grade 3 or 4 anemia in 10 patients (12.8%) in our study group vs 0 patients in NETTER-1, grade 3 or 4 leukopenia in 10 patients in our study group vs 1 patient (0.9%) in NETTER-1, and grade 3 or 4 thrombocytopenia in 7 patients (9.0%) in our study group vs 2 patients (1.8%) in NETTER-1.^12^ This discrepancy may be associated with the high rate of pretreament prior to PRRT initiation in our study group. While 1 patient in our study required ongoing red blood cell transfusion support, no one in our group went on to develop myelodysplastic syndrome, kidney failure requiring hemodialysis, or liver failure. An appreciation of the frequency of these laboratory-measured toxic effects during therapy is important in clinical practice, and it is reassuring that most laboratory-measured toxic effects were transient within our study period. Prolonged follow-up may be needed to better capture long-term PRRT-related toxic effects.

Measuring PRRT outcomes using PFS and overall survival may be helpful to set expectations for clinicians and patients. We found that 30 patients (38.5%) had disease progression or died during the study period. The median PFS for our study group was 21.6 months, which is similar to the results from the NETTER-1 group, which had a 65.2% estimated rate of PFS at month 20, with a 95% CI of 50.0% to 76.8%.^12^ These results are encouraging for implementation of PRRT in clinical practice, where clinicians may expect a substantially lower response to therapy compared with during the stringent conditions of clinical trials. We also found that having a small bowel primary tumor was associated with a lower rate of progression compared with having a pancreatic primary tumor. Median PFS for patients with primary pancreatic NETs was 13.3 months, whereas median PFS had not been reached in the small bowel group after 22 months. These findings suggest that in clinical practice, patients with small bowel NETs may have the best PRRT outcomes.

We assessed overall survival in our study group; however, because 68 of 78 patients in our study group (87.2%) were living at the time of analysis, we could not assess median overall survival. In statistical analysis, we found that patients diagnosed with NET at older ages had a higher risk of dying during the study period; however, the HR for increased risk was 1.08, suggesting a modest association. Studies of PRRT with longer follow-up periods in US-based cohorts may be important in better determining the association of PRRT with overall survival in clinical practice.

### Limitations

This study has several limitations. The small sample size may be underpowered to detect statistically significant differences between groups. The heterogeneity of the study group may be associated with confounding variables. Other limitations include the limited length of our follow-up period and the substantial proportion of our study population who had not completed 4 doses of PRRT by the time of analysis. Despite these limitations, we generated PRRT outcome data, which may contribute important information about the understudied clinical outcomes of PRRT in US-based NET populations. Additionally, we included all patient imaging after initiation of PRRT, which may not adequately assess tumor response to PRRT, as response may be slow and could be associated with confounding by pseudoprogression. This is a radiographic pattern that suggests progression based on RECIST criteria that may be associated with transient PRRT-induced tumor inflammation.^25^ We believe, however, that discovery of imaging-based disease progression prior to completion of PRRT may be clinically relevant, and therefore, should be included in the analysis. Additionally, our study group represents a heavily pretreated population that may not be representative of other practices around the world; however, this population may be more similar, compared with clinical trial populations, to those in other US-based NET practices.

## Conclusions

In this cohort study of patients with metastatic NETs, we found that PRRT may have favorable outcomes when administered among a diverse population of patients with NETs. While laboratory-measured toxic effects were common, they were seldom associated with serious complications during the study period. Furthermore, we found that patients with small bowel NETs had a significantly longer median PFS after PRRT compared with patients with pancreatic or other NETs. Larger and longer prospective studies are needed to further support these findings and determine how to optimally incorporate PRRT into the treatment of metastatic NETs.

## Notes

Supplement. **eAppendix.** Laboratory-measured Toxic Effects Per Common Terminology Criteria for Adverse Events Version 5.0 **eTable 1.** Univariable Analysis of Factors Associated With Progression Among Patients With Reviewable Imaging After Treatment **eTable 2.** Univariable Analysis of Factors Associated With Survival After Treatment **eFigure.** Overall Survival After Treatment
