# Supplementary material for: Laboratory, Clinical, and Survival Outcomes Associated With Peptide Receptor Radionuclide Therapy in Patients With Gastroenteropancreatic Neuroendocrine Tumors
Source: JAMA Netw Open. 2021 Mar 23;4(3):e212274. doi: 10.1001/jamanetworkopen.2021.2274 (PMC7988364; doi:10.1001/jamanetworkopen.2021.2274)
Supplement: Supplement. — eAppendix. Laboratory-measured Toxic Effects Per Common Terminology Criteria for Adverse Events Version 5.0 eTable 1. Univariable Analysis of Factors Associated With Progression Among Patients With Reviewable Imaging After Treatment eTable 2. Univariable Analysis of Factors Associated With Survival After Treatment eFigure. Overall Survival After Treatment [file jamanetwopen-e212274-s001.pdf]

Supplemental Online Content

Kipnis ST, Hung M, Kumar S, et al. Laboratory, clinical, and survival outcomes associated with peptide receptor radionuclide therapy in patients with gastroenteropancreatic neuroendocrine tumors. *JAMA Netw Open*. 2021;4(3):e212274. doi:10.1001/jamanetworkopen.2021.2274

eAppendix. Laboratory Measured Toxic Effects Per Common Terminology Criteria for Adverse Events Version 5.0
eTable 1. Univariable Analysis of Factors Associated With Progression Among Patients With Reviewable Imaging After Treatment
eTable 2. Univariable Analysis of Factors Associated With Survival After Treatment
eFigure. Overall Survival After Treatment

This supplemental material has been provided by the authors to give readers additional information about their work.

© 2021 Kipnis ST et al. *JAMA Network Open.*

eAppendix. Laboratory Measured Toxic Effects Per Common Terminology Criteria for Adverse Events Version 5.0[1]

Leukopenia, anemia, and thrombocytopenia were defined as the new development of a WBC count less than 3000/mm^3, Hgb less than 10g/dL, and Plt count less than 75,000/mm^3 respectively. Nephrotoxicity was defined as the new development of an eGFR less than 60mL/min/1.73m^2. Biochemical liver injury was defined as the new development of a total bilirubin greater than 1.5x the upper limit of normal, AST greater than 3x the upper limit of normal, or ALT greater than 3x the upper limit of normal.

Grade 3/4 toxicities were defined as WBC count less than 2000/mm^3, Hgb less than 8g/dL, and Plt count less than 50,000/mm^3 respectively. Grade 3/4 nephrotoxicity was defined as the new development of an eGFR less than 30mL/min/1.73m^2. Grade 3/4 biochemical liver injury was defined as the new development of a total bilirubin greater than 3x the upper limit of normal, AST greater than 5x the upper limit of normal, or ALT greater than 5x the upper limit of normal.

© 2021 Kipnis ST et al. *JAMA Network Open.*

eTable 1. Univariable Analysis of Factors Associated With Progression Among Patients With Reviewable Imaging After Treatment

	HR (95% CI)	P-value
Age at diagnosis (years), median (IQR)	1.01 (0.97-1.05)	0.58
Age at first PRRT (years), median (IQR)	0.99 (0.95-1.03)	0.68
Male sex	0.82 (0.37-1.82)	0.63
NET primary location		
Pancreas	REFERENCE	
Small bowel	0.21 (0.07-0.59)	**<0.01**
Other/unknown	0.58 (0.24-1.43)	0.24
Grade of NET		
1	REFERENCE	
2	1.22 (0.44-3.36)	0.70
3	3.07 (0.86-11.00)	0.09
Systemic therapy prior to PRRT	1.09 (0.49-2.44)	0.83
Liver-directed therapy prior to PRRT	0.71 (0.32-1.58)	0.40
Hepatic resection	1.02 (0.42-2.45)	0.97
Radiofrequency ablation	0.73 (0.10-5.44)	0.76
Bland embolization	0.76 (0.22-2.55)	0.65
TACE	0.70 (0.24-2.07)	0.52
TARE	0.91 (0.31-2.67)	0.87
Prior resection of primary	0.76 (0.32-1.79)	0.53

© 2021 Kipnis ST et al. *JAMA Network Open.*

eTable 2: Univariable Analysis of Factors Associated With Survival After Treatment

	HR (95% CI)	P-value
Age at diagnosis (years), median (IQR)	1.08 (1.01-1.16)	**0.02**
Age at first PRRT (years), median (IQR)	1.07 (0.99-1.15)	0.10
Male sex	1.96 (0.49-7.85)	0.34
NET primary location		
Pancreas	REFERENCE	
Small bowel	1.25 (0.11-13.78)	0.86
Other/unknown	6.39 (0.77-53.19)	0.09
Grade of NET		
1	REFERENCE	
2	1.47 (0.27-8.01)	0.66
3	1.58 (0.14-17.43)	0.71
Systemic therapy prior to PRRT	+	1.00
Liver-directed therapy prior to PRRT	0.49 (0.13-1.82)	0.28
Hepatic resection	0.33 (0.04-2.67)	0.30
Radiofrequency ablation	1.38 (0.17-11.04)	0.76
Bland embolization	1.58 (0.33-7.64)	0.57
TACE	0.44 (0.05-3.51)	0.44
TARE	0.64 (0.08-5.10)	0.67
Prior resection of primary	0.48 (0.13-1.79)	0.27

© 2021 Kipnis ST et al. *JAMA Network Open.*

eFigure. Overall Survival After Treatment
OS for the entire cohort of individuals with metastatic NETs who underwent PRRT.

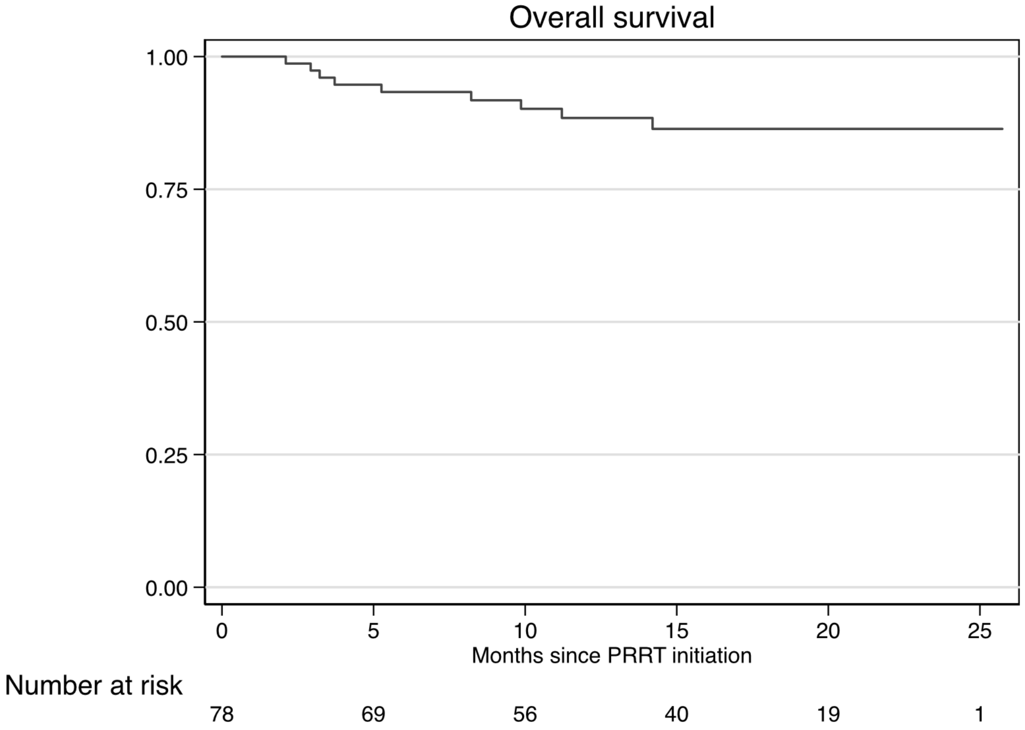

© 2021 Kipnis ST et al. *JAMA Network Open.*

Supplemental Content References

1. Common Terminology Criteria for Adverse Events (CTCAE) Version 5.0.

November 27, 2017. Accessed October 12, 2020.

https://ctep.cancer.gov/protocolDevelopment/electronic_applications/ctc.htm - ctc_50

© 2021 Kipnis ST et al. *JAMA Network Open.*
